# Reproducibility of Functional Connectivity and Graph Measures Based on the Phase Lag Index (PLI) and Weighted Phase Lag Index (wPLI) Derived from High Resolution EEG

**DOI:** 10.1371/journal.pone.0108648

**Published:** 2014-10-06

**Authors:** Martin Hardmeier, Florian Hatz, Habib Bousleiman, Christian Schindler, Cornelis Jan Stam, Peter Fuhr

**Affiliations:** 1 Department of Neurology, Hospital of the University of Basel, Basel, Switzerland; 2 Swiss Tropical and Public Health Institute, University of Basel, Basel, Switzerland; 3 Department of Clinical Neurophysiology and Magnetoencephalography, VU University Medical Center, Amsterdam, The Netherlands; University of British Columbia, Canada

## Abstract

Functional connectivity (FC) and graph measures provide powerful means to analyze complex networks. The current study determines the inter-subject-variability using the coefficient of variation (CoV) and long-term test-retest-reliability (TRT) using the intra-class correlation coefficient (ICC) in 44 healthy subjects with 35 having a follow-up at years 1 and 2. FC was estimated from 256-channel-EEG by the phase-lag-index (PLI) and weighted PLI (wPLI) during an eyes-closed resting state condition. PLI quantifies the asymmetry of the distribution of instantaneous phase differences of two time-series and signifies, whether a consistent non-zero phase lag exists. WPLI extends the PLI by additionally accounting for the magnitude of the phase difference. Signal-space global and regional PLI/wPLI and weighted first-order graph measures, i.e. normalized clustering coefficient (gamma), normalized average path length (lambda), and the small-world-index (SWI) were calculated for theta-, alpha1-, alpha2- and beta-frequency bands. Inter-subject variability of global PLI was low to moderate over frequency bands (0.12<CoV<0.28), higher for wPLI (0.25<CoV<0.55) and very low for gamma, lambda and SWI (CoV<0.048). TRT was good to excellent for global PLI/wPLI (0.68<ICC<0.80), regional PLI/wPLI (0.58<ICC<0.77), and fair to good for graph measures (0.32<ICC<0.73) except wPLI-based lambda in alpha1 (ICC = 0.12). Inter-electrode distance correlated very weakly with inter-electrode PLI (−0.06<rho<0) and weakly with inter-electrode wPLI (−0.22<rho<−0.18). Global PLI/wPLI and topographic connectivity patterns differed between frequency bands, and all individual networks showed a small-world-configuration. PLI/wPLI based network characterization derived from high-resolution EEG has apparently good reliability, which is one important requirement for longitudinal studies exploring the effects of chronic brain diseases over several years.

## Introduction

Functional connectivity (FC), graph and nodal network measures are powerful tools to characterize brain function in healthy subjects as well as in neurological and psychiatric diseases [1], [2], [3]. Based on the concept of the brain as a large complex network of interconnected elements [4], different brain regions interact in the resting state as well as in response to a stimulus or task by synchronization of oscillatory activity [5], [6]. Besides structural and functional MRI, magneto- and electro-encephalography (MEG/EEG) have been used to determine FC [7], [8].

Scalp signals of EEG are an admix of source activity, volume conduction, i.e. the spatial spread of the electric field during its way from its source through the cerebro-spinal fluid and skull [9], and the influence of the reference electrode [10]. These latter two properties may artificially induce FC as the same signal is measured at different electrodes [11]. In order to circumvent these problems, measures as the imaginary coherence [11] and the phase-lag-index (PLI) [8] have been proposed. The FC estimation by the PLI is based on a consistent lag between the instantaneous phases of two electrodes and is less sensitive to zero-lag phase-relations typical for common sources. The weighted PLI (wPLI) is an extension to the PLI and is reported to be less sensitive to noise [12].

Graph theory provides metrics to characterize complex networks [2], [13]. Based on the functional connectivity matrix, indices of functional segregation and integration have been established [14]. Two basic measures are the clustering coefficient describing the connectedness of direct neighbors of a node and the minimum path length describing the average minimal distance of a node to all other nodes in the network. The ratio between the mean normalized clustering coefficient and mean normalized path length indicates whether a network displays an efficient small-world-configuration; i.e. a combination of high local connectedness and short paths to all other nodes in the network minimizing costs for information processing [15].

In order to be useful for characterizing disease states and for capturing disease progression, FC estimates and graph measures should have low inter-subject variability and high test-retest-reliability (TRT) in healthy controls. Only few studies reported on these properties so far, mainly at short-term retest intervals of several weeks. Using MEG and mutual information (MI) as the measure of FC, Deuker et al. [16] reported good TRT for FC and moderate to good TRT for graph measures in the delta to beta-band during an n-back task and considerably lower TRT during an eyes-open resting state condition. Also using MEG and MI, Jin et al. [17] found moderate to good TRT for nodal network measures in eyes-open and eyes-closed resting state, respectively.

The current study reports on the inter-subject variability and long-term test-retest-reliability of the PLI and the wPLI (PLI/wPLI) derived from high-resolution eyes-closed resting state EEG and of first-order graph measures in the signal-space. Additionally, the relation between inter-electrode distance and PLI/wPLI is explored to empirically probe susceptibility to volume conduction; furthermore, the PLI/wPLI connectomes are displayed.

## Material and Methods

### Subjects

The study was approved by the local ethics committee (Ethikkommission beider Basel, Basel; Switzerland; EK 74/09), and all participants gave written informed consent before study inclusion. At baseline, 48 healthy subjects (median age: 36.0 years, range: 20.0–49.5; female: 73%) were examined. Inclusion criteria comprised unremarkable personal history, normal neurological exam and an EEG-recording without pathological alterations as judged from clinical EEG reading; no concurrent medical treatment was allowed. Four subjects had to be excluded from analysis due to artifactual or low-voltage EEG signal. Thirty-five subjects had a follow-up after one and two years with technically satisfying EEGs.

### EEG recording

Subjects were seated comfortably in a reclining chair in a dimly lit, sound attenuated and electromagnetically shielded room. They were instructed to relax, but to stay awake and to minimize eye and body movements. A continuous EEG during an eyes-closed resting state condition was recorded for 12 min with a 256-channel EEG system (Netstation 200 with HydroCel Geodesic Sensor Net, Electrical Geodesics, Inc., Oregon, USA). The electrode net was placed with Fz, Cz, Oz, and the preauricular points as landmarks. Electrode impedances were kept below 40 kOhm. Recording band-pass was 0.1–100 Hz, sampling frequency 1 kHz, and the vertex was used as the recording reference. During data acquisition, a subset of electrodes was monitored online to check for vigilance and artifacts by a technician. Inter-electrode distances were calculated based on a template electrodes cap with dimensions 15.3×19.5×19.3 cm.

### EEG processing

Several semi-automated, visually controlled pre-processing steps were employed using customized MATLAB code optimized for epoch selection in resting-state EEG (TAPEEG, https://sites.google.com/site/tapeeg/ [18]). In brief, all EEG were first visually inspected by an experienced neurophysiologist (MH) and segments of 25 to 200 sec containing the least amount of artifacts and sleepiness were selected. Data of 214 electrodes (excluding cheek and neck electrodes) were filtered (0.5–70 Hz; high order least-squares filter) and automatic detection of bad channels using Faster- and Fieldtrip-algorithms [19], [20] was applied (median number of interpolated channels per subject: 1, range: 0–3). Thereafter, the EEG was decomposed by independent component analysis (EEGLAB; [21]) and reconstructed after excluding components loading on the electro-cardiogramm, line noise in single electrodes or single gross artifacts; at maximum 5% of components were excluded. For epoch selection, the EEG was re-referenced to average reference, bad channels were interpolated using spherical splines [22] and a combined segment of at least 120 sec length was created; at intersections an inverse hanning window was applied. By a second visual inspection, remaining periods of drowsiness and artifacts as well as intersections were labeled as “bad”. Finally, an automatic epoch selection was performed in which one second periods labeled as “bad” (manually or by algorithm) had a very low probability to be included into a final epoch. Based on previous results, twelve 4-sec-epochs were used for further analysis, as they have been shown to be more reliable than four 12-second-epochs of identical total length in the same dataset [23].

### Measures of functional connectivity

The phase-lag-index (PLI; [8]) and the weighted PLI (wPLI; [12]) were used as measures of functional connectivity and were calculated using TAPEEG [18]. Shortly, the PLI is an index of the asymmetry in the distribution of phase differences calculated from the instantaneous phases of two time-series, here the signal of a pair of electrodes:
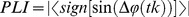
(1)


ΔΦ is the phase difference at time-point k between two time series and is determined for all time-points (k = 1 … N) per epoch (N = 4096), sign stands for signum function, <> denotes the mean value and || indicates the absolute value. Instantaneous phases were determined by the Hilbert transformation, applying a Hanning window on the concurrent fast Fourier transform. PLI ranges between 0 and 1. Common sources as volume conduction and the reference electrode do not generate a phase-lag between the time-series of two electrodes, thus phase differences center around 0 or +/− π, resulting in a PLI near or equaling 0; time-series without coupling (“noise”) generate a symmetric uniform phase distribution also resulting in a PLI near or equaling 0. In contrast, a consistent phase-lag between two time-series generates an asymmetric distribution of phase differences reflecting true interactions, and a completely asymmetric distribution results in a PLI of 1.

The wPLI is an extension of the PLI [12]. By weighing each phase difference according to the magnitude of the lag, phase differences around zero only marginally contribute to the calculation of the wPLI. This procedure reduces the probability of detecting “false positive” connectivity in the case of volume conducted noise sources with near zero phase lag and increases the sensitivity in detecting phase synchronization [12].Weighing is achieved by using the imaginary component of the cross-spectra as a factor. We employ here the debiased wPLI estimator according to formulas 26 and 32 in Vinck et al. [12].

For further analysis, PLI/wPLI was first calculated for each pair of electrodes per epoch based on N = 4096 phase difference vectors, thereafter twelve replicates were averaged to generate the average PLI/wPLI weight matrix per subject.

Analysis was done on a global and on a regional level of spatial resolution. Global PLI/wPLI equals the average of all PLI/wPLI values of the average weight matrix per subject. Regional PLI/wPLI is based on 22 anatomically defined regions comprising 7 or 8 electrodes (n = 170, excluding electrodes in the midline and at the outer border, see Figure S1). For each region, the average connectivity of all its electrodes to all other regional groups of electrodes was determined, i.e. the regional degree (row average of respective electrodes of the weight matrix). In addition, the connectivity between each two regions was calculated (average over cells of the weight matrix belonging to respective electrodes of two regions) resulting in n = 231 links. For correlation to distance, PLI/wPLI values of pairs of electrodes and their respective inter-electrode distances were used. To display the connectomes, the grand means over all average PLI/wPLI weight matrices at baseline were plotted. PLI/wPLI was calculated for the theta-(4–8 Hz), alpha1-(8–10 Hz), alpha2-(10–13 Hz) and beta-(13–30 Hz) band using a butterworth bandpass-filter.

### Graph measures

Graph measures were calculated based on the average PLI/wPLI weight matrix of the twelve epochs per subject in each frequency band (n = 214 nodes). Regional weight matrices (n = 22 nodes) were not used, as it is disputable whether graph measures in small networks are meaningful [24]. Calculation of graph measures on each single epoch weight matrix and subsequent averaging had resulted in lower test-retest reliability (see Table S1). This is probably due to the fact that averaging the single epoch weight matrices diminishes momentary connectivity patterns and spurious connectivity due to noise resulting in the individual “core” connectivity. However, momentary connectivity patterns and subsequent network characterization by graph measures may show different aspects than the network characterization based on the average connectivity matrix. In order to avoid arbitrary thresholds and unconnected nodes, weighted network analysis was employed in which each edge is equivalent to the measured PLI/wPLI of two interconnected nodes. Undirected measures of functional segregation and integration were calculated according to the definitions given in Stam et al. [25]; respective formulas were implemented in TAPEEG [18].

The weighted clustering coefficient C quantifies the intensities of the subgraphs of a node and is equivalent to the unweighted clustering coefficient normalized by the average intensities of triangles at the node, if the weight matrix is symmetric and weights ranging between 0 and 1 [25], [26]. The weighted clustering coefficient at node *i* is defined as:
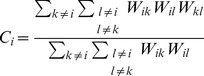
(2)in which *w* is defined as the weight between two nodes. The average over all C_i_ is the mean clustering coefficient (Cw), a global measure of functional segregation of the network [14], [27].

The weighted shortest path length L_ij_ gives the average of the shortest distances of one node to each other node in the network, where shortest distance in the weighted case is defined as the smallest inverse of the sum of PLI values of connecting edges between *i* and *j* if *w*_ij_ unequals zero, and L_ij_ is infinity if *w*_ij_ equals zero. The average over all L_ij_ is the weighted average path length (Lw), a global measure of functional integration of the network [14], [28] and is defined as:
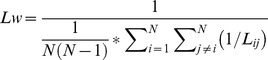
(3)in which *N* is defined as the number of nodes in the network. Using the harmonic mean instead of the arithmetic mean handles infinitive path lengths from unconnected nodes [25].

In order to make graph measures independent of network size and better comparable between subjects, they were normalized [25]. Edge weights of an original network were randomly reshuffled preserving network size but destroying network structure, and Cw and Lw were calculated for this random network. Using the average Cw and Lw of 50 surrogate random networks iterated five times in the denominator and Cw and Lw in the nominator, the normalized Cw or *gamma* and the normalized Lw or *lambda* were calculated.

To determine whether networks show a small-world-configuration, the small-world-index (SWI; [29]) was calculated as the ratio between gamma and lambda for each subject. An index >1 signifies efficient small-world topology, i.e. the combination of high local clustering, as typical for regular networks and short path length, as typical for random networks; small-world topology has been shown to be a salient feature of many real-world networks [15] including the human brain [30].

### Statistical analysis

Cross-sectional inter-subject variability was expressed as the coefficient of variation (CoV) calculated as the ratio between the standard deviation and the mean of global PLI/wPLI, gamma, lambda and SWI at baseline. TRT over three time points was estimated for the same measures as well as for regional degree and regional links using the intra-class-correlation coefficient (ICC [3], [1]; [31]). A bootstrapping procedure with replacements and 10000 permutations was performed to estimate the 95% confidence interval (95% CI) for both indices. In accordance with previous studies, TRT was categorized as “excellent” if ICC>0.75, as “good” if ICC: 0.60–0.75, as “fair” if ICC: 0.40–0.60 and as “poor” if ICC<0.40 [16], [17], [32].

Spearman's rank correlation coefficient was used to measure associations between inter-electrode distance and PLI/wPLI within subjects. ANOVAs were used to compare global PLI/wPLI values between frequency bands at baseline and within frequency bands between time points. The topographies of the mean connectivity distribution (connectome) were compared between frequency bands by using the average nodal degree over all subjects at baseline in permutation tests on ANOVA with frequency band as factor. Permutation statistics were used to control for multiple comparisons and non-Gaussian distributions [33].

## Results

Inter-subject variability of global PLI was low to moderate over frequency bands (0.12<CoV<0.28; Table 1) and very low for PLI based gamma, lambda and SWI (CoV<0.022, CI 95%: 0.01–0.027). Global wPLI showed higher inter-subject variability (0.25<CoV<0.55, Table 1) but comparable values for wPLI based graph measures (CoV<0.048, CI 95%: 0.012–0.059).

**Table 1:** Inter-subject variability of global PLI and wPLI at baseline by frequency band expressed by the coefficient of variation (CoV; CI: confidence interval estimated from bootstrapping).

		theta	alpha1	alpha2	beta
**PLI**		**0.12**	**0.23**	**0.28**	**0.15**
	95% CI	0.08–0.20	0.17–0.31	0.21–0.38	0.12–0.17
**wPLI**		**0.25**	**0.44**	**0.55**	**0.29**
	95% CI	0.14–0.41	0.33–0.56	0.39–0.76	0.25–0.33

TRT was good to excellent for global PLI over frequency bands (0.68<ICC<0.79), and moderate to good for PLI-based graph measures (gamma: 0.48<ICC<0.65; lambda: 0.51<ICC<0.73; SWI: 0.33<ICC<0.63; see Table 2). Global wPLI had comparable TRT (0.70<ICC<0.80) but lower values for wPLI-based graph measures (gamma: 0.43<ICC<0.57; lambda: 0.12<ICC<0.47; SWI: 0.32<ICC<0.51; Table 3).

**Table 2:** Test-retest-reliability of global PLI and PLI-based graph measures over time by frequency band expressed by the intraclass-correlation coefficient (ICC; CI: confidence interval estimated from bootstrapping; SWI: small-world-index).

		theta	alpha1	alpha2	beta
**PLI**		**0.72**	**0.79**	**0.74**	**0.68**
	95% CI	0.49–0.92	0.69–0.90	0.63–0.87	0.46–0.81
**gamma**		**0.65**	**0.64**	**0.48**	**0.58**
	95% CI	0.43–0.85	0.39–0.88	0.24–0.67	0.36–0.76
**lambda**		**0.73**	**0.57**	**0.56**	**0.51**
	95% CI	0.52–0.90	0.23–0.87	0.32–0.73	0.18–0.80
**SWI**		**0.56**	**0.63**	**0.33**	**0.56**
	95% CI	0.34–0.79	0.40–0.86	0.16–0.60	0.40–0.69

**Table 3:** Test-retest-reliability of global wPLI and wPLI-based graph measures over time by frequency band expressed by the intraclass-correlation coefficient (ICC; CI: confidence interval estimated from bootstrapping; SWI: small-world-index).

		theta	alpha1	alpha2	beta
**wPLI**		**0.78**	**0.80**	**0.74**	**0.70**
	95% CI	0.56–0.94	0.69–0.90	0.63–0.88	0.50–0.83
**gamma**		**0.57**	**0.43**	**0.49**	**0.53**
	95% CI	0.33–0.81	0.14–0.78	0.21–0.70	0.28–0.75
**lambda**		**0.47**	**0.12**	**0.41**	**0.38**
	95% CI	0.27–0.66	−0.02–0.30	0.14–0.62	0.11–0.64
**SWI**		**0.49**	**0.50**	**0.32**	**0.51**
	95% CI	0.21–0.75	0.24–0.81	0.11–0.55	0.38–0.65

On the regional level, ICC values are given as medians of the 22 ICC values per regional degrees and 231 ICC-values per inter-regional links over frequency bands. PLI had good TRT for regional degree (0.58<ICC<0.75) and fair to good TRT for inter-regional links (0.42<ICC<0.61; Table 4). TRT of wPLI were comparable for regional degree (0.59<ICC 0.77) and inter-regional links (0.41<ICC<0.64; Table 5). Figure S2 and Figure S3 show the topographic distribution of ICC values per frequency band and electrode for PLI/wPLI, respectively.

**Table 4:** Test-retest-reliability of regional PLI over time by frequency band.

		theta	alpha1	alpha2	beta
**regional degree**	**median**	**0.66**	**0.75**	**0.71**	**0.58**
	range	0.51–0.78	0.57–0.83	0.63–0.76	0.42–0.64
**inter-regional links**	**median**	**0.47**	**0.61**	**0.60**	**0.42**
	range	0.08–0.83	0.24–0.85	0.41–0.79	0.01–0.82

The median (range) intraclass-correlation coefficients over 22 regional degrees and 231 inter-regional links are given.

**Table 5:** Test-retest-reliability of regional wPLI over time by frequency band.

		theta	alpha1	alpha2	beta
**regional degree**	**median**	**0.71**	**0.77**	**0.72**	**0.59**
	range	0.57–0.84	0.60–0.85	0.62–0.78	0.49–0.67
**inter-regional links**	**median**	**0.53**	**0.64**	**0.61**	**0.41**
	range	0.14–0.84	0.23–0.87	0.34–82	0.05–0.79

The median (range) intraclass-correlation coefficients over 22 regional degrees and 231 inter-regional links are given.

The correlation of inter-electrode distance with inter-electrode PLI was very weak in all subjects at all frequency bands (median rho over frequency bands: −0.06<rho<0) with no clear direction, albeit highly significant in single subjects (maximal positive correlation: rho  = 0.15, maximal negative correlation: rho = −0.19; p<0.0001) due to the high number of values (214*213/2 data points per subject). For inter-electrode wPLI, a weak negative correlation to inter-electrode distance was found (median rho over frequency bands: −0.22<rho<−0.18) and in single subjects maximal negative correlation was rho = −0.4, maximal positive correlation was rho  = 0.03, p<0.0001).

**Figure 1:**
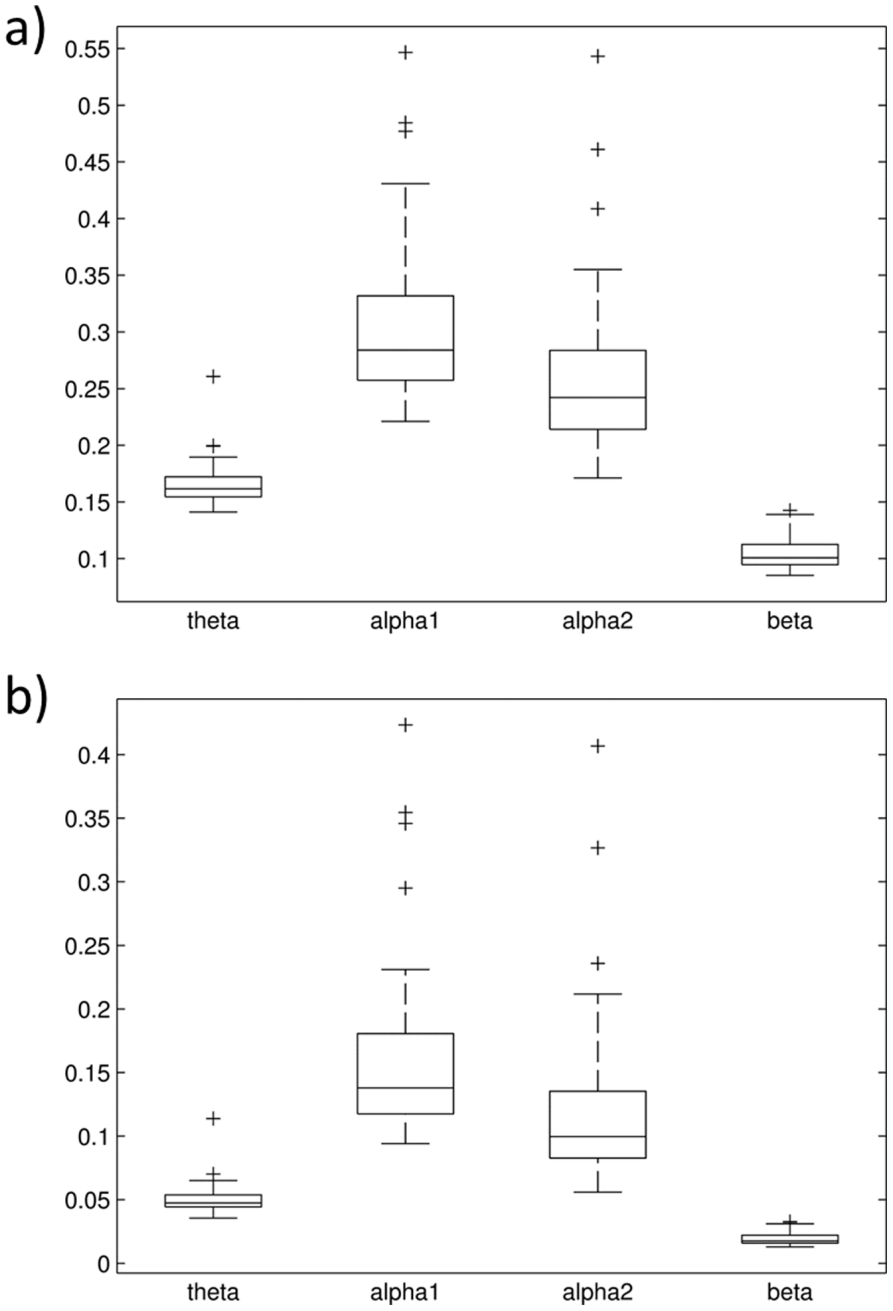
Distribution of a) global PLI values and b) global wPLI values between subjects at baseline in different frequency bands; all bands are significantly different (see text).

**Figure 2:**
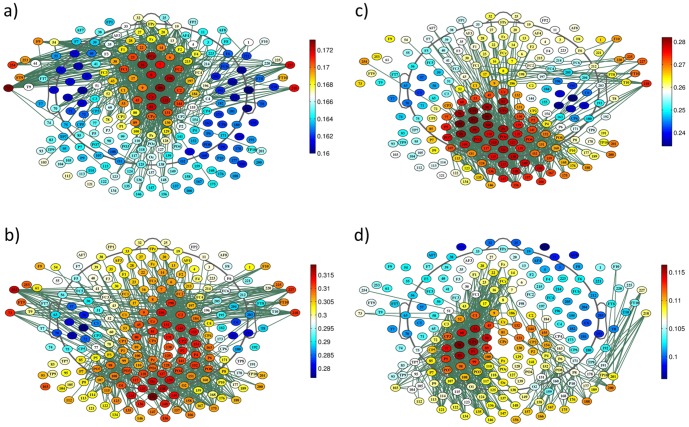
Topographic plots of the grand mean PLI connectomes (green: 3% strongest links are plotted) and grand mean PLI value per electrode (nodal degree, values are color-coded) over all subjects at baseline by frequency band: a) theta, b) alpha1, c) alpha2, d) beta.

**Figure 3:**
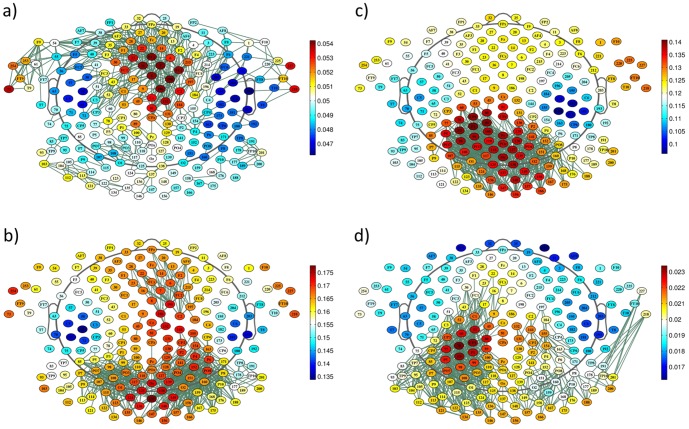
Topographic plots of the grand mean wPLI connectomes (green: as in Figure 2) and grand mean wPLI value per electrode (color-codes as in Figure 2): a) theta, b) alpha1, c) alpha2, d) beta.

Global PLI and wPLI were significantly different between frequency bands at baseline (PLI: F = 127, p<0.0001; wPLI: F = 75, p<0.0001; Figure 1a and 1b); in post-hoc t-tests, all bands were significantly different to each other (p<0.05 for alpha1 vs. alpha2, p<0.0001 for all other comparisons). Within frequency bands global PLI/wPLI showed no significant differences over time (PLI: F<0.45, p>0.5; wPLI: F<0.33, p>0.5). Topographic connectivity patterns differed significantly between frequency bands at the single electrode level, i.e. in nodal degree (PLI: F>73, p_corrected_<0.001; wPLI: F>45, p_corrected_<0.001; respective connectomes are displayed in Figure 2 and Figure 3 and in Figure S4 and Figure S5 for regional connectomes).

All individual networks showed a small-world-configuration (medians over frequency bands; PLI-based SWI: 1.024<SWI<1.029, range: 1.016–1.078; wPLI-based SWI: 1.053<rho<1.069, range: 1.017–1.200).

## Discussion

Characterization of functional connectivity by PLI shows good to excellent long-term test-retest-reliability over two years, and mainly good long-term test-retest-reliability of PLI-based graph measures; inter-subject variability is acceptably low. Functional connectivity determined by the wPLI shows comparable TRT as the PLI, wPLI-based graph measures are slightly less reliable and inter-subject variability is higher. High-resolution EEG is a suitable recording modality when care is taken that the measure of functional connectivity is not relevantly influenced by common sources as volume conduction and the reference electrode. The weak negative correlation between inter-electrode distance and inter-electrode wPLI is presumably due to the weighing factor, as short range connections are more likely to have large consistent phase difference than long range connections; volume conduction defined as zero-lag phase difference is neither detected by PLI nor by wPLI.

Only a few studies report so far on test-retest-reliability of functional connectivity and network measures, mainly at short-term test-retest intervals of several weeks. In fMRI-studies using correlations between BOLD-signal time series to estimate functional connectivity, test-retest-reliability was only moderate in one study [34], and even low to poor in another [32]. Using MEG and mutual information as the measure of functional connectivity, Deuker et al. [16] found good test-retest-reliability for FC as well as several global graph measures during an eyes-open n-back task at a test-retest interval of 6–8 weeks. In higher frequency bands (beta- and gamma-band), during eyes-open resting state and in second-order graph measures as for example the small-world-index, the TRT was comparably lower. Using as well MEG and mutual information, Jin et al. [17] reported fair to moderate TRT for different nodal centrality measures at a test-retest-interval of two weeks, which was partly higher in the eyes-open as compared to the eyes-closed resting state, and much lower in the gamma-band.

In the current study, beta-band TRT as well as small-world-index TRT also tends to be lower compared to lower frequency bands and first-order graph-measures, respectively. As pointed out previously this is probably due to the different physiological function as higher frequencies may serve to establish cognitive representation, whereas lower frequency bands are more anatomically constrained [35], [36]. The gamma-band has not been studied here due to its sensitivity to muscle artifacts, which partly also applies for the beta-band [37]. The level of spatial resolution on which connectivity is determined influences the TRT with highest TRT on the global level and slightly lower TRT for regional degree. On the level of inter-regional links TRT is highly variable ranging from poor for some links to good and excellent in others. Regional connectivity analysis may, on the one hand, better catch more localized group differences in for example parietal hub regions than global measures, and, on the other hand, is more robust to slight variations of local maxima and outliers in connectivity compared to analyses on the single electrode level.

Several methodological differences may explain the higher TRT of eyes-closed resting state reported in the current study as compared to previous studies. First, MEG mainly picks up signals from sources within sulci, whereas the EEG-signal is mostly driven by sources on the gyri [38], [39]; second, mutual information depicts a different aspect of connectivity than measures based on phase synchronization as the PLI and wPLI [7], and third, the way of band-pass-filtering, bandwidth and the choice of unweighted [16] or weighted networks ([17], current study) may play a critical role.

For chronic brain diseases evolving over years, long-term TRT as shown in the current study for the PLI/wPLI is paramount since ageing may influence networks as well [40], [41]. Whether networks constitute a stable trait over many years up to a certain age in analogy to the spectral “fingerprint” of the EEG [42], [43] and as suggested by a stable association of genetic features and functional connectivity [44], [45] remains to be elucidated. However, in a small group of elderly healthy controls around 60 years, the PLI-based normalized clustering coefficient decreased in the alpha-bands over a four year period, whereas path length and other frequency bands did not change significantly [46].

The current study does not allow conclusions on the validity of the PLI/wPLI and PLI/wPLI-based graph measures derived from EEG with respect to characterization and monitoring of diseases of the central nervous system. Currently it is far from clear how tight measures of functional connectivity, which express a mere statistical interdependency [47], are associated with the known thalamo-cortical and other networks involved in the generation of oscillatory brain activity [36]. In particular the resting state scalp signal is difficult to interpret, albeit several studies have shown a complex relationship between resting state brain oscillations and resting state networks derived from functional MRI [48], [49]. However, PLI/wPLI networks show a clear dominance in connectivity in parieto-occipital regions, corresponding to the topography of structural and functional connectomes derived from MRI studies [30], [50]. Additionally, they show clear differences between frequency bands as would be expected by the known physiological differences between frequency bands in spectral analysis [51], [52], [53], [54], [55] and as noted in previous MEG studies [17], [56]. Smallworldness, a feature apparent in PLI/wPLI-based networks in the current study is more difficult to interpret; in particular as weighted network analysis was used [24]. Furthermore, the concept of smallworldness is challenged by a recent anatomical study [57] and has even been reported to be artificially induced in EEG-model data [58] (see below). However, PLI and PLI-based graph measures have been shown to differentiate between healthy controls and patients with Alzheimer's diseases (AD) [25], Parkinson's disease [46], Multiple Sclerosis [59], and even demonstrated group differences in a clinical trial on a medical food in AD [60]. The wPLI has only been applied in rodent local field potentials showing clear differences in a task related paradigm [12].

Volume conduction is one of the main methodological problems in functional connectivity studies using EEG or MEG [9], [11]. The PLI has explicitly been developed to be insensitive to zero-lag phase differences [8], which are a hallmark of volume conduction. In the current study, there was no consistent relation between PLI and inter-electrode distance, confirming robustness against volume conduction. However, using signal modeling Peraza et al. [58] report that volume conduction may influence the PLI when multiple independent sources are present and in turn biases graph measures. The study compared unweighted networks based on 64 uncorrelated sources to networks based on the same sources multiplied by a forward solution to simulate volume conducted scalp signal. Both models were expected to generate random networks but this was not true for the simulated scalp signal and even small-worldness was found [58]. To reduce such spurious connectivity due to uncorrelated noise, Vinck et al [12] proposed the wPLI. Still, in real data, PLI/wPLI may detect both, physiological and spurious connectivity, in particular on the single subject level. Averaging over epochs reduces noise but is constrained by the availability of a sufficient number of good quality epochs. Applying rigorous ICA filtering by selecting only components of interest harbours the risk to exclude important information systematically. However, given all these caveats, several studies have shown that resting state connectivity and graph measures differ between healthy controls and patients with brain disease [25], [46], [59], [60]. Another relevant limitation of the PLI/wPLI is the downside of its insensitivity to volume conduction: physiological connectivity with zero phase-lag may remain undetected, and thus, PLI/wPLI may underestimate short-range connections and, in terms of networks, local segregation or clustering [56].

The influence of the recording electrode can be greatly diminished by re-referencing to the average of all electrodes when using high-resolution EEG with 128 or more electrodes, as in a closed system the average signal sums up to zero [9]. Another way to deal with common sources would be the reconstruction of the signal in the source space; however, the translation matrix may itself induce artificial functional connectivity [61] and methodology is only going to be developed [56].

Regarding quantification of TRT, the intra-class correlation coefficient is widely used but has been criticized as being susceptible to bias [62], [63], as it is only a relative index of reliability and can be inflated by few subjects with high within-subjects variability. Using the bootstrapping technique to estimate 95% confidence intervals, we partly diminished this bias. Still, comparisons to studies not indicating confidence intervals remain difficult, as the ICC-value alone may only be a rough estimate of TRT.

## Conclusions

PLI/wPLI based network characterization derived from high-resolution EEG-recordings is apparently reliable over two years on a global and regional level of spatial resolution. Which physiological mechanisms are exactly reflected by these measures in the resting state is currently far from clear, but beyond the scope of the current study. However, good long-term test-retest-reliability is one important requirement for a biomarker. Network characterization may help to explore the effects of chronic disorders on the functional organization of the brain. Long-term TRT in older subjects in whom effects of ageing may have more impact remains to be studied. As high-resolution EEG is widely available and easy to administer data may even be gathered in a multicenter setting, allowing to reach appropriate sample sizes for testing hypotheses on functional reorganization in brain diseases in due time.

## Supporting Information

Figure S1 **Topography of groups of electrodes used for regional analysis.**
(PDF)

Figure S2 **Topography of test-retest-reliability (ICC) of mean PLI per electrode (nodal degree).**
(PDF)

Figure S3 **Topography of test-retest-reliability (ICC) of mean wPLI per electrode (nodal degree).**
(PDF)

Figure S4 **Connectomes of regional PLI.**
(PDF)

Figure S5 **Connectomes of regional wPLI.**
(PDF)

Table S1 **Test-retest-reliability of graph measures calculated in single weight matrices.**
(PDF)
