# Supplementary material for: Reproducibility of Functional Connectivity and Graph Measures Based on the Phase Lag Index (PLI) and Weighted Phase Lag Index (wPLI) Derived from High Resolution EEG
Source: PLoS One. 2014 Oct 6;9(10):e108648. doi: 10.1371/journal.pone.0108648 (PMC4186758; doi:10.1371/journal.pone.0108648)
Supplement: Figure S1 — Topography of groups of electrodes used for regional analysis. (PDF) [file pone.0108648.s001.pdf]

Fig S1; Groups of electrodes used for regional analysis

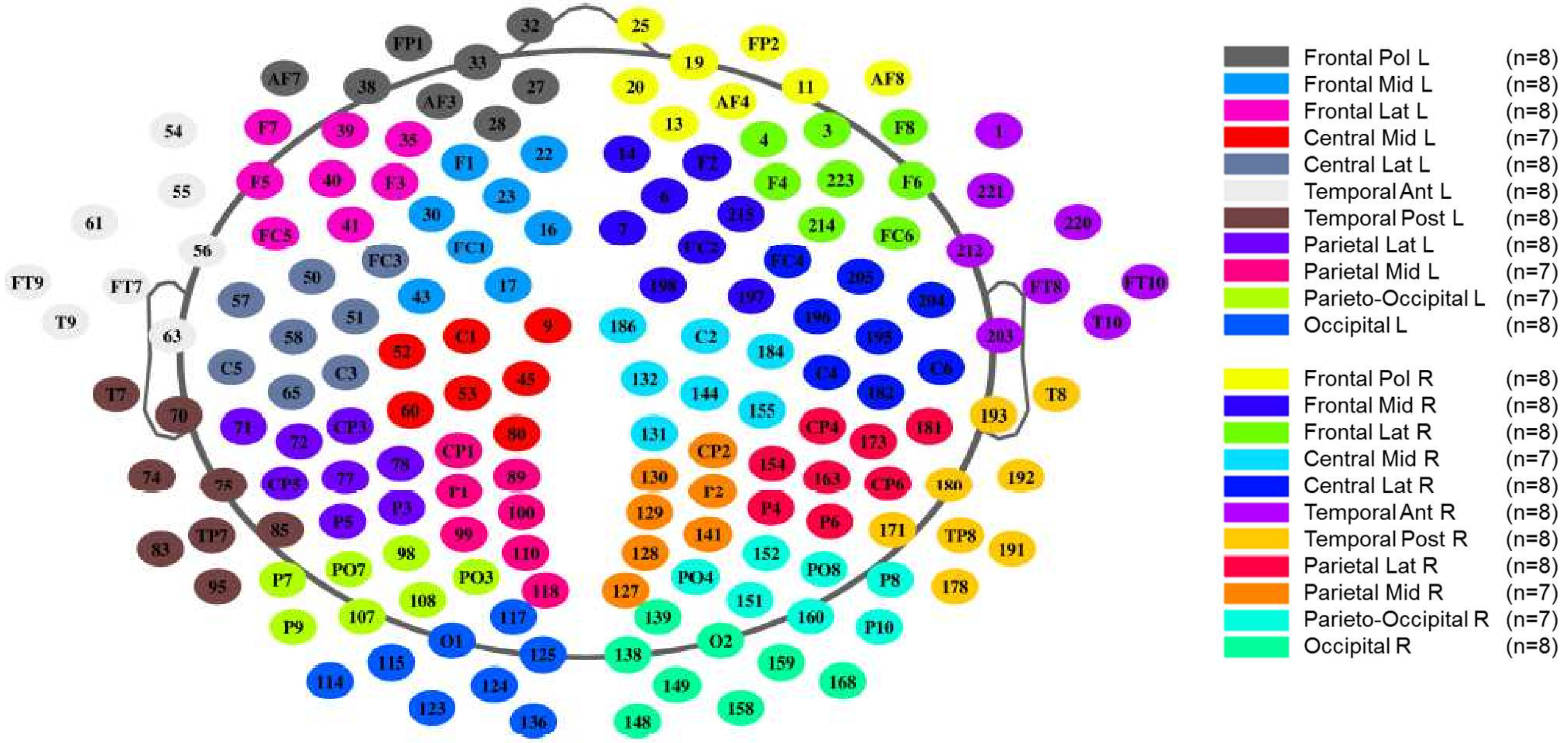

Fig S1. Groups of electrodes averaged for regional analysis (n=170); midline electrodes (n=12) and electrodes at the very border of the 214-electrode array (n=16 per hemisphere) were excluded from regional analysis
