# Supplementary material for: Reproducibility of Functional Connectivity and Graph Measures Based on the Phase Lag Index (PLI) and Weighted Phase Lag Index (wPLI) Derived from High Resolution EEG
Source: PLoS One. 2014 Oct 6;9(10):e108648. doi: 10.1371/journal.pone.0108648 (PMC4186758; doi:10.1371/journal.pone.0108648)
Supplement: Figure S3 — Topography of test-retest-reliability (ICC) of mean wPLI per electrode (nodal degree). (PDF) [file pone.0108648.s003.pdf]

Fig S3; Topography of test-retest-reliability of mean wPLI per electrode

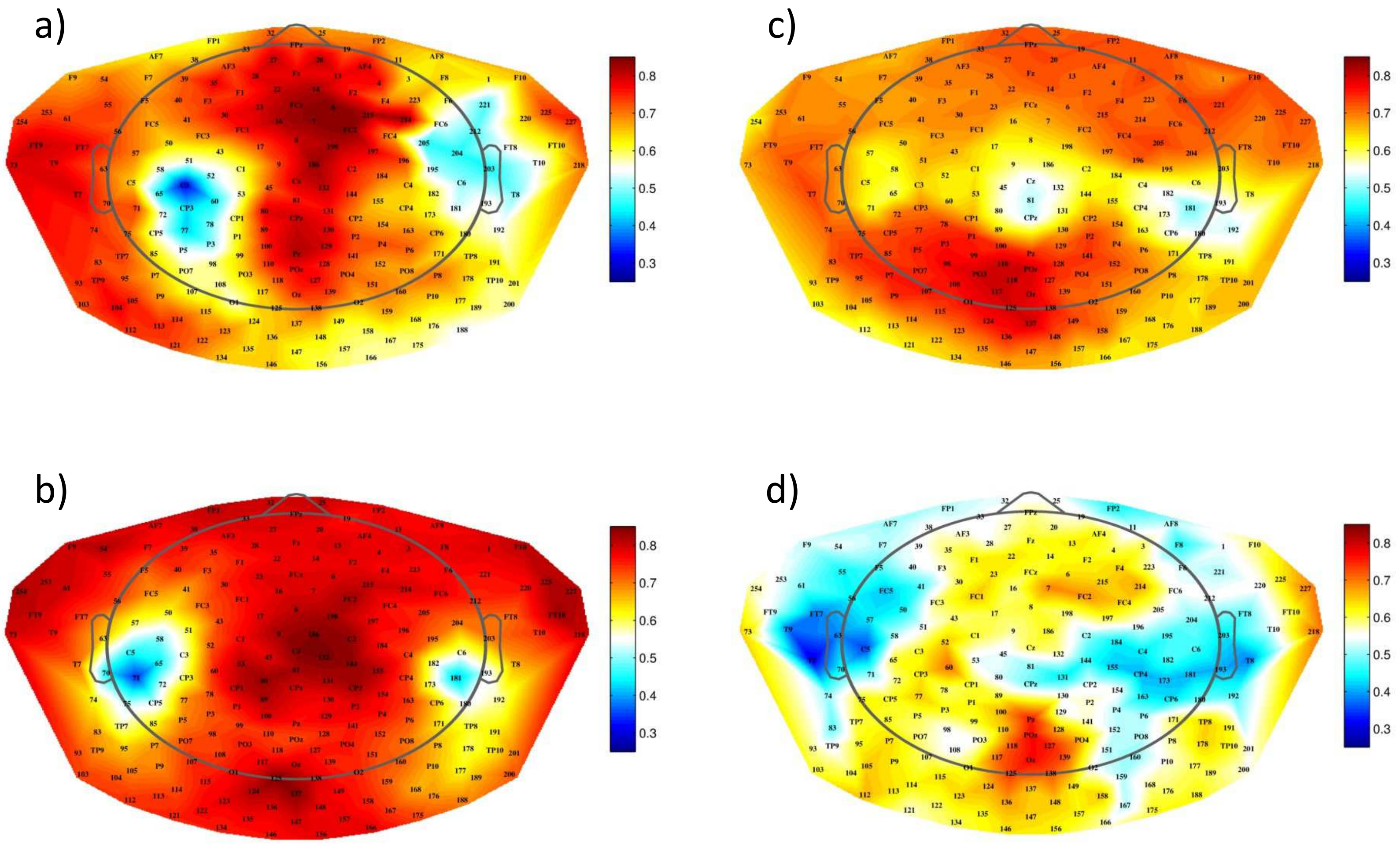

Fig S3. Topography of test-retest-reliability of mean wPLI per electrode (nodal degree); ICC values (range: 0.25 to 0.85) are plotted by frequency band: a) theta-, b) alpha1-, c) alpha2-, d) beta-band.
