# Supplementary material for: Reproducibility of Functional Connectivity and Graph Measures Based on the Phase Lag Index (PLI) and Weighted Phase Lag Index (wPLI) Derived from High Resolution EEG
Source: PLoS One. 2014 Oct 6;9(10):e108648. doi: 10.1371/journal.pone.0108648 (PMC4186758; doi:10.1371/journal.pone.0108648)
Supplement: Figure S4 — Connectomes of regional PLI. (PDF) [file pone.0108648.s004.pdf]

Fig S4; Connectomes of regional PLI

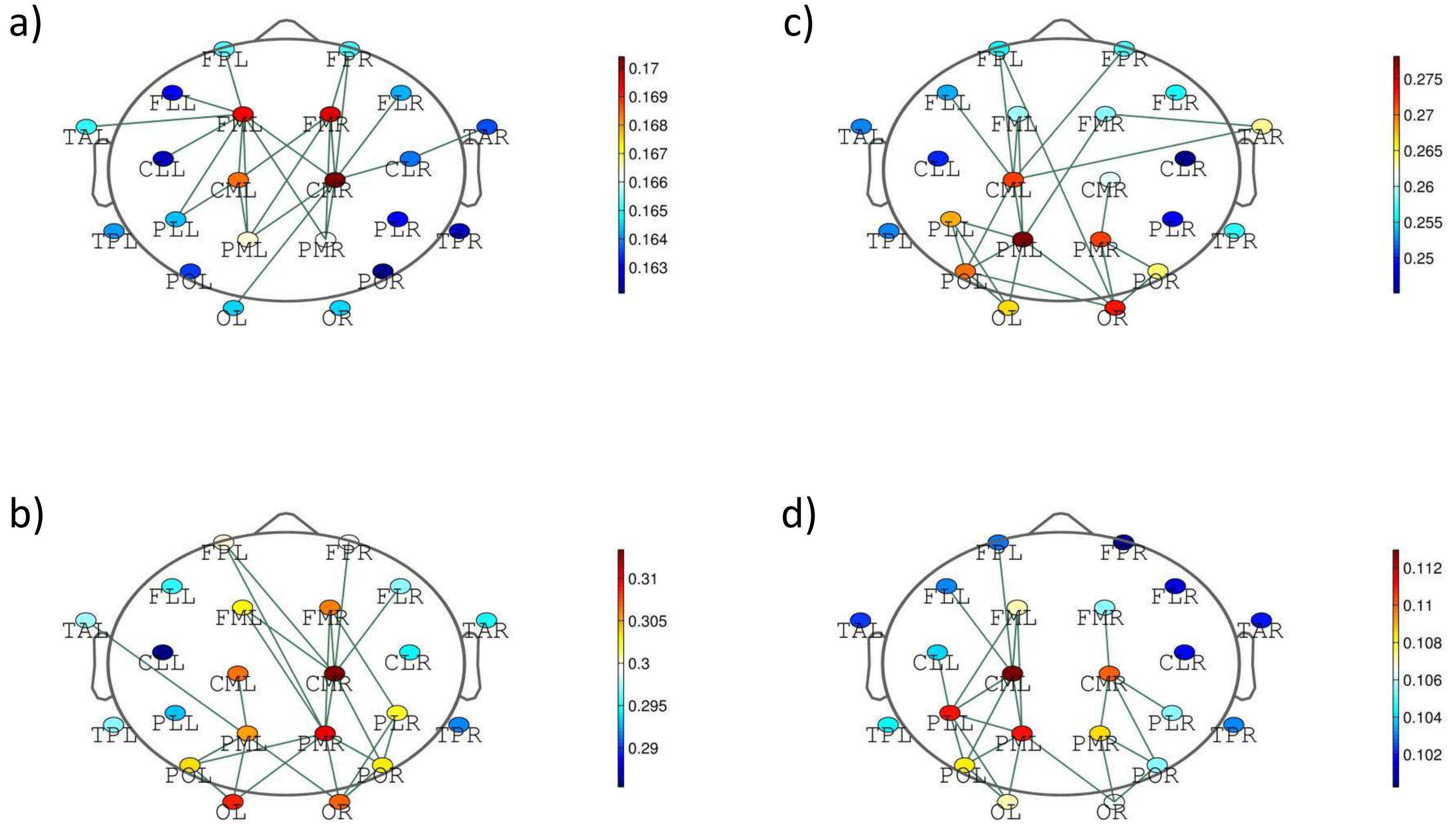

Fig S4. Topographic plots of the regional grand mean PLI connectomes (10% strongest links are plotted) and grand mean PLI per region (regional degree) over all subjects at baseline by frequency band: a) theta, b) alpha1, c) alpha2, d) beta.
