# Supplementary material for: Reproducibility of Functional Connectivity and Graph Measures Based on the Phase Lag Index (PLI) and Weighted Phase Lag Index (wPLI) Derived from High Resolution EEG
Source: PLoS One. 2014 Oct 6;9(10):e108648. doi: 10.1371/journal.pone.0108648 (PMC4186758; doi:10.1371/journal.pone.0108648)
Supplement: Table S1 — Test-retest-reliability of graph measures calculated in single weight matrices. (PDF) [file pone.0108648.s006.pdf]

Table S1 Test-retest-reliability of PLI-based graph measures when network measures are analysed in 12 single weight matrices per subject before averaging (in contrast to calculating graph measures only once per subject on the average weight matrix, see Table 2). ICC-values per frequency band with 95% confidence intervalls (CI) are given.

	theta	alpha1	alpha2	beta
**gamma**	**0.25**	**0.61**	**0.43**	**0.38**
95% CI	0.04-0.43	0.40-0.81	0.29-0.62	0.13-0.69
**lambda**	**0.50**	**0.55**	**0.58**	**0.60**
95% CI	0-32-0.67	0.28-0.71	0.39-0.79	0.43-0.74
**SWI**	**0.28**	**0.56**	**0.42**	**0.31**
95% CI	0.11-0.53	0.37-0.78	0.24-0.63	0.14-0.50
